# The Future of Minimally Invasive Capsule Panendoscopy: Robotic Precision, Wireless Imaging and AI-Driven Insights

**DOI:** 10.3390/cancers15245861

**Published:** 2023-12-15

**Authors:** Miguel Mascarenhas, Miguel Martins, João Afonso, Tiago Ribeiro, Pedro Cardoso, Francisco Mendes, Patrícia Andrade, Helder Cardoso, João Ferreira, Guilherme Macedo

**Affiliations:** 1 Precision Medicine Unit, Department of Gastroenterology, São João University Hospital, 4200-427 Porto, Portugal; miguel.pedro96@gmail.com (M.M.); joaoafonso28@gmail.com (J.A.); tiagofcribeiro@outlook.com (T.R.); pedromarilio@gmail.com (P.C.); francisco.cnm@gmail.com (F.M.); anapatriciarandrade@gmail.com (P.A.); hc@sapo.pt (H.C.); guilhermemacedo59@gmail.com (G.M.); 2 WGO Gastroenterology and Hepatology Training Center, 4200-047 Porto, Portugal; 3 Faculty of Medicine, University of Porto, 4200-427 Porto, Portugal; 4 Department of Mechanic Engineering, Faculty of Engineering, University of Porto, 4200-065 Porto, Portugal; jferreira@fe.up.pt; 5 DigestAID—Digestive Artificial Intelligence Development, 455/461, 4200-135 Porto, Portugal

**Keywords:** capsule endoscopy, panendoscopy, artificial intelligence, bioethics, green endoscopy

## Abstract

**Simple Summary:**

The exponential growth in artificial intelligence development, particularly its application in capsule endoscopy, serves as a compelling model for gastroenterologists. This review focusses on the latest advancements in capsule endoscopy, analyzing the possible benefits and ethical challenges that artificial intelligence may bring to the field of minimally invasive capsule panendoscopy, while also offering insights into future directions. Specifically in the context of oncological gastrointestinal screening, there is still a need to explore alternative strategies for enhancing this process. Artificial intelligence-enhanced capsule panendoscopy has the potential to positively impact the future by addressing time constraints and improve accessibility through the use of highly efficient diagnostic models.

**Abstract:**

In the early 2000s, the introduction of single-camera wireless capsule endoscopy (CE) redefined small bowel study. Progress continued with the development of double-camera devices, first for the colon and rectum, and then, for panenteric assessment. Advancements continued with magnetic capsule endoscopy (MCE), particularly when assisted by a robotic arm, designed to enhance gastric evaluation. Indeed, as CE provides full visualization of the entire gastrointestinal (GI) tract, a minimally invasive capsule panendoscopy (CPE) could be a feasible alternative, despite its time-consuming nature and learning curve, assuming appropriate bowel cleansing has been carried out. Recent progress in artificial intelligence (AI), particularly in the development of convolutional neural networks (CNN) for CE auxiliary reading (detecting and diagnosing), may provide the missing link in fulfilling the goal of establishing the use of panendoscopy, although prospective studies are still needed to validate these models in actual clinical scenarios. Recent CE advancements will be discussed, focusing on the current evidence on CNN developments, and their real-life implementation potential and associated ethical challenges.

## 1. Introduction to Panendoscopy and Its Challenges

Capsule endoscopy (CE) is a minimally invasive procedure that was initially conceived for evaluation of the small bowel and has achieved a high diagnostic yield for the detection of small bowel lesions [1]. The notion of a panenteric examination (e.g., for Crohn’s disease assessment) emerged with the development and implementation of colon capsule endoscopy [2]. In fact, since CE allows for the evaluation of the whole gastrointestinal (GI) tract, the concept of a single minimally invasive panendoscopy has become quite a tempting idea [3]. Technical feasibility and expected favorable patient tolerance both support the use of this method. Nevertheless, there are several challenges in implementing it.

Firstly, the implementation of capsule panendoscopy (CPE) would further increase the reading burden of an already time-consuming exam. Without any auxiliary procedural automation, this would most likely reduce the cost-effectiveness of a gastroenterology department, not to mention that many medical institutions would lack the experience or resources required to perform it [4]. More importantly, by considerably increasing the number of frames that must be reviewed, fatigue and monotony levels would increase, potentially leading to missed lesions and/or decisive frames.

Secondly, the diagnostic accuracy of CE in assessing the esophagus and stomach is still suboptimal. In addition to the inability to inflate the lumen, which is an inherent constraint of CE in any anatomical area, there are other limitations that must be considered. In the esophagus, the capsule moves quickly, especially if taken in a sitting/orthostat position, which can reduce the number of mucosal frames and may be associated with incomplete visualization of the Z-line [5]. In the stomach, which is not a cylindric structure as is the case for the small bowel, some areas, particularly proximal ones, may be overlooked, since it is entirely dependent on peristaltic motions, even when dual-headed endoscopic capsules are used [6].

Lastly, while adequate bowel preparation is one of the most important current concerns of capsule enteric evaluation, it becomes much more determinant in the scenario of CPE. In fact, we have yet to find an effective and reproducible method of bowel preparation that is widely accepted and tolerated by patients, not only for small bowel CE, but also for colon CE [7]. Even though numerous studies have been conducted in this domain, including systematic reviews with meta-analysis, it remains challenging to reach a final conclusion due to heterogeneity in how researchers analyze mucosa cleansing [8,9]. There is currently no method that fulfils the criteria of the method being non-time-consuming, consistent, and free of inter-observer variability. Neither the development of operator-dependent nor color-intensity-based automated methods have fully addressed this issue [7,8,9]. The development of a standardized method and its integration in CE reading tools most likely needs to be the former step, thus facilitating the subsequent design of an appropriate clinical trial to determine the most beneficial preparation.

## 2. Wireless Capsule Endoscopy: A Pill-Sized Revolution in Gastrointestinal Imaging

Single-camera capsules were the first to be developed in the early 2000s, initially with lower resolution and a lower capturing frame rate [1]. Over time, improvements were made, including to the camara resolution capturing frame rate and battery power, and software refinement as well as hardware advancements took place with the introduction of real-time viewers [10]. The progress eventually led to development of adaptative frame rate technology, where the faster the capsule progresses, the higher the capture rate, reaching a maximum of six frames per second [10].

Dual-camera capsules were introduced in 2006 [2]. First-generation designs went into sleep mode shortly after ingestion due to power saving issues, rewiring only in the small bowel. The capturing frame rate was poor, resulting in a lower sensitivity in detecting polyps, compared to second-generation models [2,11]. These later devices became accessible later in 2009, offered a wider view angle and came with an adaptative frame rate up to 35 frames per second, which was a valuable inclusion to preserve battery [11]. More recently, in 2016, a third-generation design was introduced which was able to stay operational without interruption along the entire GI tract [12]. Initially it was intended to assess inflammatory bowel disease patients more accurately, but it rapidly prompted discussions of CPE.

Since its introduction, CE has established itself as the first-line method for assessing the small bowel mucosa. The two main indications for its usage include suspected mid-gastrointestinal bleeding and diagnosis/follow-up in situations of suspected/confirmed small bowel Crohn’s disease [13]. Moreover, it can also be used to monitor hereditary polyposis syndromes, mainly Peutz–Jeghers, and to rule out small intestine tumors [13]. It is also applicable to the evaluation of nonresponsive or refractory celiac disease cases, when the diagnosis of celiac disease is uncertain, or in malabsorption syndromes [13]. Additionally, dual-camera capsules have improved the visualization of the colonic mucosa, by enabling greater visibility of both haustra and areas located behind folds [11]. As a result, they have improved capsules’ overall diagnostic yield, not only for detecting protuberant lesions, but also for other mucosal lesions [11]. Consequently, this has become a possible alternative method for colorectal cancer (CCR) screening, mostly in situations where prior colonoscopy was incomplete or there was a greater risk of complications or contraindication to conventional colonoscopy or sedation [14,15,16].

CE is generally safe and well tolerated, with few contraindications. Caution is warranted in patients with swallowing disorders, due to risk of aspiration [17]. Additionally, it requires clinical assessment of the risk of capsule retention [13,18]. This is particularly applicable for patients with established Crohn’s disease (ECD), where the risk of retention is increased, and whenever obstructive symptoms are observed [13,19]. Given the high risk of CE retention in Crohn’s disease, the inability to distinguish high-risk from low-risk patients based on clinical presentation alone, and the indisputable effectiveness of patency testing, the safest approach would be to pursue patency testing before CE in all ECD patients [13,19,20]. Moreover, there is also an increased risk of retention in patients with previous gastrointestinal surgery or radiation therapy of the abdomen and pelvis, as well as persistent users of non-steroid anti-inflammatories and patients with a personal history of small bowel tumors [17]. In these cases, a patency capsule might also be considered [17,21]. The use of CE in individuals with implantable cardiac devices (pacemaker, defibrillators and left ventricular assist devices) should not be contraindicated, since several studies have shown that is safe [22].

## 3. Robotic-Assisted Panendoscopy: Advancements and Benefits

**Figure 1:**
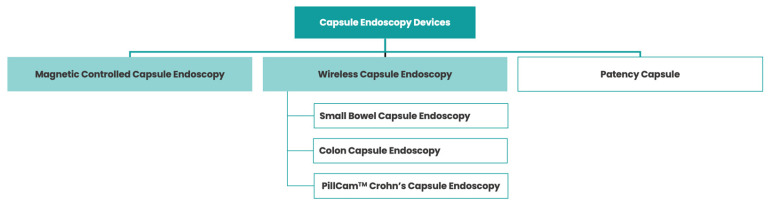
Various types of capsule endoscopy devices.

In addition to wireless CE, magnetically controlled capsule endoscopy (MCE) has emerged as an alternative method to evaluate the superior GI tract (Figure 1) [23]. In this case, the capsule contains a magnet that can be moved in real time by a magnetic field that is generated outside the patient after swallowing it, using forces of translation and rotation [24]. 

There are three types of magnetic control systems: hand-held magnets, electromagnetic coil systems (comparable to present-day MRIs) and robotic arms [25,26,27,28]. Of these techniques confined to very few centers, the latter is the most widely used and studied, mainly for the assessment of the gastric mucosa, given its operability (either manually or automatically), tolerability (the exam is conducted without patient movement) and ease of installation (compared to the installation of larger electromagnetic coil systems) [23]. 

The development and implementation of MCE for gastric assessment addresses one of the shortcomings of wireless CE by not depending entirely on stomach peristalsis to move. Although the protocol is not fully established, patients are typically asked to drink 1 L of water (generally mixed with an anti-foaming agent) 10 min prior to the start of the exam, to enhance gastric distention [28]. Then, the capsule is mobilized through this water interface, enabling evaluation of the gastric mucosa. In fact, there is some evidence that shows that MCE’s diagnostic accuracy for detecting gastric lesions might be comparable to the gold standard upper endoscopy, with superior overall agreement in 90% of cases [27]. This, in turn, may serve as a safe and effective alternative for gastric assessment, besides wireless CE, in patients who cannot tolerate esophagogastroduodenoscopy.

Furthermore, the implementation of MCE controlled by a robotic arm could also contribute to panendoscopic evaluation of the whole GI tract. For example, a patient could ingest the capsule lying down (to maximize the assessment of the esophageal mucosa), followed by an extensive evaluation protocol of the stomach with the help of magnetic fields [28]. Then, when the capsule enters the duodenum, the patient would be able to leave the examination bed and move as in wireless CE, allowing for the remaining panenteric assessment. 

When it comes to contraindications, they are similar to those outlined previously in wireless CE. The presence of a magnetic field adds extra contraindications, comparable to those applied to MRI, namely the presence of implanted electronic devices, non-MRI-compatible pacemakers and/or magnetic metal foreign bodies [28].

From a diagnostic standpoint, it should be highlighted that MCE’s ability to evaluate the fundus is still incomplete, with some studies reporting impossibility in 20% of instances [29]. Furthermore, thus far, it is challenging to compare wireless CE and MCE, given the lack of comparative research between them. 

## 4. Artificial Intelligence in Panendoscopy: Enhancing Diagnostic Accuracy

In recent years, artificial intelligence (AI) has gained relevance in diverse fields of medical practice, particularly is specialties with a strong imaging and diagnostic component [30]. Gastroenterology has always been marked by ground-breaking achievements, using highly innovative technologies to improve patient care. As a result, it is not surprising that it is also leading the way in the advancement of AI technologies in healthcare.

AI-related developments have been achieved in two areas of computational science over the previous decade: machine learning (ML) and deep learning (DL). These two fields emerged around the same period. However, the lack of adequate computational power in the past limited the widespread adoption of DL models. As a result, technology initially embraced ML algorithms. Their aim was to complete a task by analyzing patterns automatically. Nevertheless, ML requires a supervised phase to ensure proper data annotation [31]. 

With the current availability of ample computational resources, DL models have gained significant momentum in recent years. These models are a subset of machine learning that are also used for automatic pattern identification but, unlike the former, do not necessarily require human interaction to train the model, displaying supervised or unsupervised learning potential [32,33]. They involve neural networks with multiple layers (three or more), structured in a hierarchical human-brain-inspired architecture, which is capable of performing more complicated tasks by sequentially combining inputs from various layers ranging from lower-level to higher-levels ones [34]. One DL model example is a convolutional neural network (CNN), which, as the name suggests, has a multilayer neural network structure that is used to automatically analyze complex visual data, mimicking the neurobiological process [35]. 

There are some ML-based capsule software add-ons which assist the gastroenterologist in image-pattern analysis. They were developed for many purposes, including color image analysis (e.g., automatically detecting blood, as in PillCam’s Suspected Blood Indicator), topographic segmentation (e.g., automatically recognizing distinct anatomical sections) and video adjustment (e.g., reducing duration of a video by displaying frames with the highest probability of being abnormal, as in PillCam’s Top 100) [36]. These tools helped to reduce the reading burden, although the percentage of missing lesions is higher compared to that in developed DL models [37]. Therefore, there has been exponential interest in the development and validation of DL models for CE. Table 1 provides an overview of the published work regarding CNN development for CE.

Convolutional neural networks were initially developed using frames from a single-camera capsules, later expanding to dual-camera capsules. Specific CNNs were applied first in the small bowel, followed by the colon and rectum, and then, in both anatomical regions, excelling at detecting a particular lesion [37,38,39,40,41,42,43,44,45,46,47,48,49,50,51,52,53,54]. Nevertheless, adopting a sequential approach where each specific CNN is applied one at a time for an AI-assisted review of a CE video, while logical, might not be the most efficient strategy.

Complex CNNs have started to emerge, offering the capability of detecting multiple types of lesions at once [55,56,57,58,59]. The first paper in this field was published by Ding et al. and demonstrates the potential of a CNN-based approach to assisting in the reading of wireless CE. Indeed, their AI system provides the simultaneous detection of a wide range of lesions. Despite the novelty of being the first published complex model, the findings of this study are currently a topic of debate, as this CNN can accurately detect various types of lesions but fails to differentiate between them [55]. The CNN described in that study serves as the core technology for the newly developed DL solution (ProScan™, AnX Robotica, Plano, TX, USA) to be incorporated into the reading software of the NaviCam SB system (AnX Robotic Corp, Plano, TX, USA). Although the hardware has received clearance by the Food and Drug Administration (FDA), this clearance has not been granted for ProScan™, which currently awaits approval for commercial use. Other groups have also developed DL models which are most often used in the small bowel, but are also capable of being used in the colon [56,57,58,59]. 

From panenteric AI-enhanced mucosa evaluation, some groups have also tried to develop DL solutions for assessing the stomach. First, they used MCE capsules [60,61]. Then, there was also a published CNN that used various types of wireless CE capsules, representing another important step for pursuing the AI-enhanced panendoscopy vision [62].

The technological readiness level of these algorithms in CE is currently situated in the initial stages of development, spanning from experimental to demonstration pilots, with some still in the research phase focused on concept validation. To fully understand the potential of AI during CE, prospective and multicentric studies are still required since most research conducted so far has been retrospective. The role of this DL-based technology in the identification of esophageal lesions by CE is still to be explored. CE is associated with a scarcity of esophagus images, which limits the establishment of esophageal-only databases. Nevertheless, the development of these types of models may be a pivotal step towards minimally invasive AI-enhanced CPE.

## 5. Integrating Robotic Systems and Wireless Capsules: A Synergetic Approach

As previously discussed, CPE allows for visualization of the whole GI system, particularly if proper bowel preparation is carried out [58]. This could be a valuable asset in evaluating cases of inflammatory bowel disease and overt GI bleeding [63,64,65]. Moreover, with AI advancements, CPE could become a cost-effective cancer screening method. Given the different types of capsules already available, it is debatable whether technology should advance towards wireless CE panendoscopy or robotic MCE panendoscopy.

From a global perspective, wireless CE is more widespread, in contrast to MCE, which is only found in very few centers currently. Although there are published CNNs for both modalities for the detection of gastric lesions, there are more groups working with AI on wireless CE. Although there are no studies comparing diagnostic performance between wireless CE and MCE, it is possible that panendoscopy based on wireless CE could be more affordable and effective. In fact, wireless panendoscopy has the potential advantage of having the capability to be performed in homecare, without the patient having to be in a clinic or hospital. 

Nonetheless, in countries with high prevalence of stomach cancer, choosing robotic panendoscopy to screen both gastric and colorectal cancer could be a reasonable approach, taking into account the MCE features previously discussed. Having available both wireless and robotic CE expands and diversifies the toolkit of minimally invasive CE. Robotic CE has the potential to address specific limitations of wireless CE, offering enhanced stomach visualization. Moreover, it could create possibilities for tissue sampling and even therapeutic interventions, given the increased control over capsule propulsion [66].

## 6. Overcoming Limitations: AI-Assisted Navigation in Panendoscopy

The implementation of ever less invasive diagnostic/therapeutic procedures has contributed to the evolution of medicine. As a result, it may be anticipated that progress will be made in the development of alternative diagnostic modalities to assess the digestive system, in addition to the currently gold standard upper and lower endoscopy. The thought process is that, whereas CE classically focuses on the small bowel, it may be capable of assessing the whole GI tract, starting from the esophagus and progressing through the stomach to the small bowel, colon and rectum.

**Figure 2:**
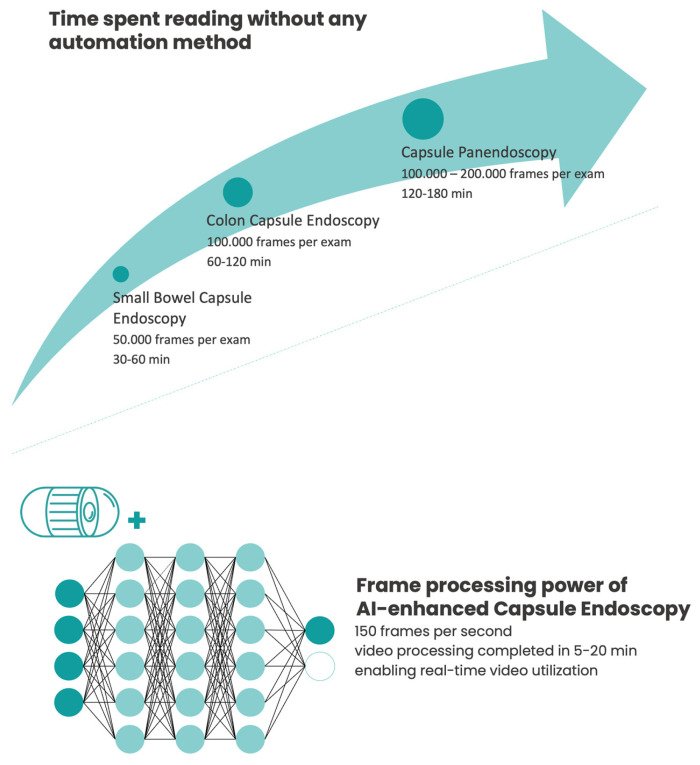
Conventional reading time vs. artificial intelligence (AI)-enhanced capsule endoscopy assessment.

CPE has the potential to change the way GI diseases are evaluated. The case of GI oncological screening is a challenge worth mentioning, since colorectal and gastric cancers are two of the top five malignancies affecting countries with a high human development index [67]. Although CE could serve as an alternative non-invasive screening method and be able to assess these two anatomical locations at once, it would be too time-consuming and would probably result in non-negligible false negative rates. In this clinical scenario, this would only be possible with the aid of AI technology, significantly reducing CPE’s health-related burden (Figure 2) [68]. 

Aside from being a procedure that consumes a considerable amount of time and incurs increased costs, it is important to note CPE’s additional constraints of being a single-use procedure and not being able to perform therapeutic interventions (can robotic CE change this?) [66]. However, despite these limitations, its noteworthy disruptive potential warrants emphasis that, in the long run, with DL technology optimization, it might be a suitable alternative (cost-effective) to opt for CPE as the preferred populational oncological screening method for the GI tract. This is based on the notion that its diagnostic accuracy would be comparable to the current gold standard, making it more likely to be accepted by most patients, as it is less invasive and does not require air inflation, radiation or sedation. Consequently, upper and lower endoscopy would mostly be used to clear diagnostic uncertainties, obtain tissue for histological and molecular analysis and treat CPE-detected lesions. 

On top of this, by adopting a more interventional approach for conventional upper and lower endoscopy, gastroenterology could work towards becoming greener [69]. In fact, this is one of pressing concern of this field, as it involves an elevated amount of single-use disposable materials and a large number of resources for adequate device disinfection [70]. If AI-assisted CPE proves to be cost-effective, then it has the potential to significantly reduce the number of unnecessary exams, particularly those with a primary diagnostic aim, lowering endoscopy’s carbon footprint.

## 7. Improving Patient Experience: Wireless Capsule Endoscopy vs. Traditional Endoscopy

The importance of upper and lower endoscopy in advancing the field of gastroenterology cannot be overstated, as they successfully combine diagnostic and therapeutic functionalities. Nonetheless, they are invasive procedures with a not insignificant complication risk [71]. Furthermore, they may cause discomfort in a proportion of patients and may even be poorly tolerated by some individuals [72].

These procedures can certainly be supplemented with sedation techniques, serving to both improve patient comfort while also empowering the gastroenterologist’s diagnostic and therapeutic proficiencies. However, these might also lead to a higher risk of complications, increase recovery times for patients and escalate costs (including the loss of working days) [72].

When compared to traditional endoscopy, CPE may improve patient experience. Patients would still need to follow a low-fiber diet for a few days, and take an oral bowel preparation whose substance, timing and dose have yet to be established and optimized [21,73]. Following the ingestion of the capsule, the patient would need to check the capsule’s transit from stomach (or to complete a robotic capsule gastric protocol), and to administer an additional booster once the capsule has reached the duodenum (0 and 3 h after) [68]. However, it is important to acknowledge that wireless CPE could be more easily incorporated into a widespread daily routine. Moreover, it can potentially decrease the reluctance of patients as is less invasive and does not require air inflation, radiation or sedation [3].

## 8. Ethical Considerations and Challenges in AI-Assisted Panendoscopy

**Figure 3:**
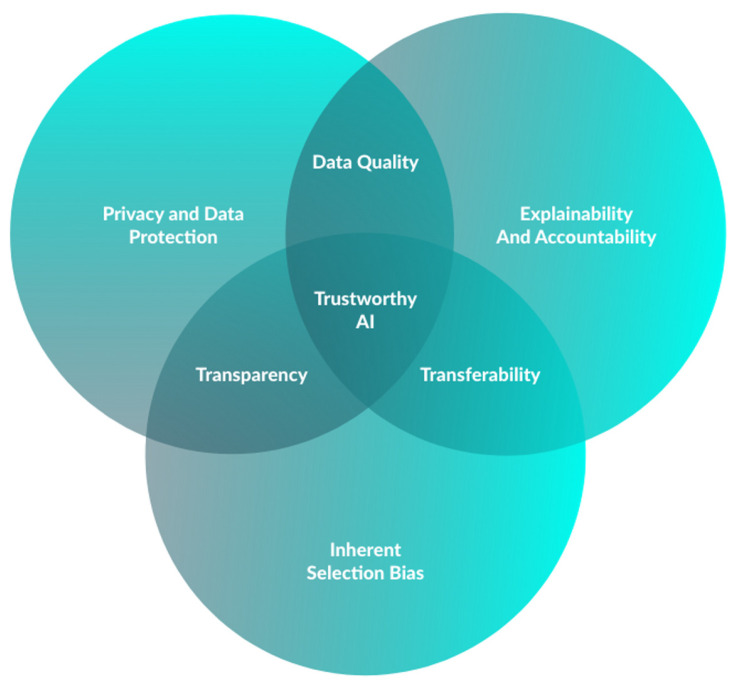
Essential criteria for development of trustworthy AI in capsule endoscopy.

AI’s widespread acceptance is dependent on addressing three main groups of bioethical challenges, encompassing data acquisition (input), model development (AI tool) and the impact of AI-generated responses on clinical practice (output) (Figure 3) [74].

In the first place, it is important to acknowledge that the process of developing a CNN is a complex test that requires the acquisition and standardization of an extensive volume of data. With information becoming more readily available and possibly collected without individuals’ knowledge, privacy concerns may arise. Moreover, as cyberattacks become more frequent, there is also an obvious need for appropriate data protection (e.g., respecting the General Data Protection Regulation 2016/79 in E.U), as well as non-traceability [75,76]. Current innovations in healthcare blockchain may mitigate these concerns, given the decentralized data framework using chronological and immutable blocks [77].

Furthermore, we must deal with the inherent selection bias present in the dataset used to train the deep learning model [78]. On the one hand, the effectiveness of an AI algorithm is directly related to the quality of its training data. If this is insufficient or inaccurate, it might lead to inappropriate CNN development [78]. On the other, even with a high-quality dataset, the model’s training population may lack proper representation, impacting its external validity [74]. In addition, extended training in one population may result in model overfitting, in which it may not yield equivalent diagnostic performance outcomes when exposed to different data [79]. Assuring data quality is a pivotal role of the medical community in AI-related research.

There are also two clinical scenarios one must consider when discussing these. The first is related to the “black-box” nature of this AI technology, since it may detect patterns (in this case, lesions) that physicians cannot notice [80]. Although this explainability problem arises in other aspects of modern medicine (e.g., drugs that improve a patient’s prognosis without knowing the exact mechanism), decisions based on AI that are not made by humans face greater resistance [74]. The second is when the model fails at detecting a relevant lesion, resulting in a false negative. In fact, there may still be an accountability problem, since the absence of a human decision will not exempt someone from taking responsibility for an untoward event [74]. 

**Figure 4:**
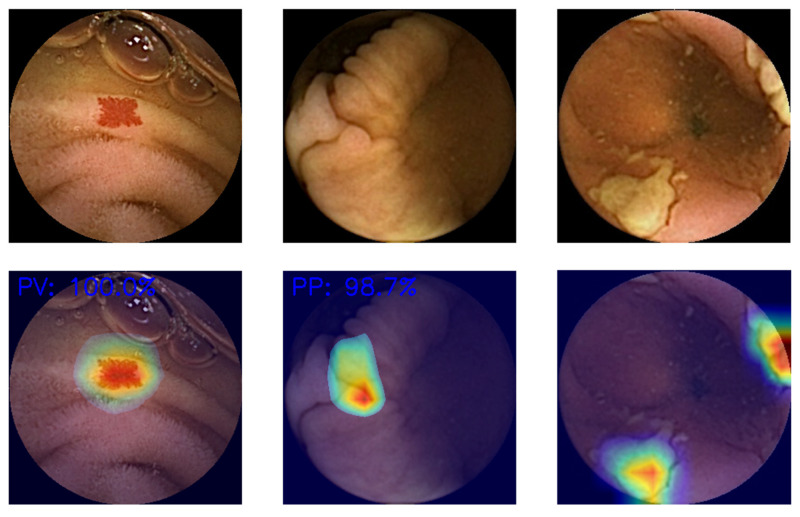
Examples of generated heatmaps.

Even if AI-assisted CE performance proved to be equivalent to that of experienced endoscopists in assessing full-length CE videos in prospective validation studies, it is still necessary to discuss which of the following modes of presentation is better: while reading the video (e.g., square lesion delimitation while the video is playing) or prior to this (e.g., DL software analyses of the full-video, selecting the most relevant frames for the physician) [81]. In the first case, the model is simpler to understand, but there is a risk of ignoring surrounding areas, and the reading time reduction would be lower. In the other, this would be a less biased approach to image assessment, although there is a higher risk of incomplete visualization of the video. Moreover, the gastroenterologist may struggle to comprehend the model’s frame selection. Emerging solutions like heatmaps may address this by delimitating the area with the maximum probability of lesion presence (Figure 4). 

AI integration in real practice requires well-regulated channels. Some technologies have previously received FDA clearance, such as AI/ML-Based Software as a Medical Device (SaMD). In general, regulation is written in such way that any changes made after the original market authorization would require premarket FDA review [82]. Nonetheless, since CNNs evolve and adapt quickly, is essential to recognize that new frameworks capable of adequately regulating them are still necessary.

## 9. Concluding Remarks—Enabling the Goal of Establishing the Use of Panendoscopy: Robotic and Wireless Capsule Endoscopy Assisted by Artificial Intelligence

The exponential growth of AI publications demonstrating excellent diagnostic accuracy, while demonstrating proficient processing power, has the potential to disrupt the current paradigm.

In a short time, gastroenterologists will possess two major tools to provide better care for patients. One is conventional endoscopy, whose therapeutic potential is pushing its traditional boundaries beyond imagination. The other involves the ongoing advancements in AI technology in this specialty. While the first factor is widely acknowledged as one of the primary factors motivating doctors to pursue it, the second one is still seen with high levels of caution.

The medical community may be concerned about the ongoing technological advancements. Still, this should be embraced as a new era, comparable to changes seen after industrialization and the emergence of the global web and search engines. The integration of AI and big data knowledge into medical professionals’ core curriculum is an important step, as well. On the one hand, doctors must partner with engineers and data scientists to craft such technology, since medical expertise is crucial to the development of valid databases. On the other, even without direct involvement in model creation, doctors should understand AI studies to know if their findings are applicable to their patients. 

Currently, the majority of studies concerning deep learning model development in CE are based on still frames or video segments. Moreover, there is still no SaMD approved by FDA that is capable of multiorgan evaluation and suitable for various devices. Conducting prospective and multicentric studies and assessing AI models with full videos, in real clinical scenarios, are a necessary steps before considering CPE’s implementation in daily routine. This milestone must be fulfilled before considering the use of AI-assisted minimally invasive CPE.

## Figures and Tables

**Table 1:** Overview of the published work regarding convolutional neural network (CNN) development for capsule endoscopy.

	Publication, Author, Year	Study Aim	Capsule Types	Centers n	Exams n	Frames n		Types of CNN	Dataset Methods	Analysis Methods	Classification Categories	Performance Metrics		
						Total	Lesion					SEN	SPE	AUC
**Specific CNN for Small Bowel Lesions**	Aoki, 2020 [37]	Detection of blood	SB2 SB3	1	66	38,055	6711	ResNet	Frame labeling of all datasets (normal vs. blood content)	Train–test split (73–26%)	Blood	97	100	100
	Afonso, 2021 [38]	Detection of blood	SB3	2	1483	23,190	13,515	Xception	Frame labeling of all datasets (normal vs. blood or hematic residues)	Staged incremental frame with train–test split (80–20%)	Blood/hematic residues	98	98	100
	Leenhardt, 2019 [39]	Detection of angiectasia	SB3	French national of still frames database (from 13 centers)	NA	1200	600	YOLO	Previous manual annotation of all angiectasias for the French national database	Deep feature extraction of dataset already manually annotated (300 lesions and 300 normal frames) Validation with classification of new dataset (300 lesions and 300 normal frames)	Angiectasia	100	96	NK
	Tsuboi, 2020 [40]	Detection of angiectasia	SB2 SB3	2	169	12,725	2725	SSD	Manual annotation of all angiectasias	CNN is trained exclusively in positive frames (2237 with angiectasias) Validation on mixed data with positive and negative frames (488 angiectasias and 10,000 normal frames)	Angiectasia	99	98	100
	Houdeville, 2021 [41]	Detection of angiectasia	SB3 Mirocam	NA	NA	12,255	613	YOLO	Previous trained on SB3 devices	Validation with 626 new SB3 still frames and 621 new Mirocam still frames	Angiectasia (SB3)	97	99	NK
											Angiectasia (Mirocam)	96	98	NK
	Ribeiro, 2021 [42]	Detection of vascular lesions + categorization of bleeding potential	SB3	2	1483	11,588	2063	Xception	Frame labeling of all datasets (normal (N) vs. red spots (P1V) vs. angiectasia or varices (P2V))	Train–test split (80–20%) with 3 × 3 confusion matrix	N vs. all	90	97	98
											P1V vs. all	92	95	97
											P2V vs. all	94	95	98
	Aoki, 2019 [43]	Detection of ulcerative lesions	SB2 SB3	1	180	15,800	5800	SSD	Manual annotation of all ulcers or erosions	CNN is trained exclusively in positive frames (5630 with ulcers) Validation on mixed data with positive and negative frames (440 lesions and 10,000 normal frames)	Ulcers or erosions	88	91	96
	Klang, 2020 [44]	Detection of ulcers + differentiation from normal mucosa	SB3	1	49	17,640	7391	Xception	Frame labeling of all datasets (normal vs. ulcer)	5-fold cross-validation with train–test split (80 vs. 20%)	Ulcers (mean of cross-validation)	95	97	99
	Barash, 2021 [45]	Categorization of severity grade of ulcers	SB3	1	NK	Random selection of 1546 ulcer frames from Klang dataset		ResNet	Frame labeling of all datasets (mild ulceration (1) vs. moderate ulceration (2) vs. severe ulceration (3))	Train–test split (80–20%) with 3 × 3 confusion matrix	1 vs. 2	34	71	57
											2 vs. 3	73	91	93
											1 vs. 3	91	91	96
	Afonso, 2021 [46]	Detection of ulcerative lesions + categorization of bleeding potential	SB3	2	2565	23,720	5675	Xception	Frame labeling of all datasets (normal (N) vs. erosions (P1E) vs. ulcers with uncertain/intermediate bleeding potential (P1U) vs. ulcers with high bleeding potential (P2U))	Train–test split (80–20%) with 4 × 4 confusion matrix	N vs. all	94	91	98
											P1E vs. all	73	96	95
											P1U vs. all	72	96	96
											P2U vs. all	91	99	100
	Saito, 2020 [47]	Detection of protruding lesions	SB2 SB3	3	385	48,091	38091	SSD	Manual annotation of all protruding lesions (polyps, nodules, epithelial tumors, submucosal tumors, venous structures)	CNN is trained exclusively in positive frames (30,584 with protruding lesions) Validation on mixed data with positive and negative frames (7507 lesions and 10,000 normal frames)	Protruding lesions	91	80	91
	Saraiva, 2021 [48]	Detection of protruding lesions + categorization of bleeding potential	SB3	1	1483	18,625	2830	Xception	Frame labeling of all data (normal (N) vs. protruding lesions with uncertain/intermediate bleeding potential (P1PR) vs. protruding lesions with high bleeding potential (P2PR))	Train–test split (80–20%) with 3 × 3 confusion matrix	N vs. all	92	99	99
											P1PR vs. all	96	94	99
											P2PR vs. all	97	98	100
**Specific CNN for Colonic Lesions**	Yamada, 2021 [49]	Detection of colorectal neoplasias	COLON2	1	184	20,717	17,783	SSD	Manual annotation of all colorectal neoplasias (polyps and cancers)	CNN is trained exclusively in positive frames (15,933 with colorectal neoplasias) Validation on mixed data with positive and negative frames (1805 lesions and 2934 normal frames)	Colorectal neoplasias	79	87	90
	Saraiva, 2021 [50]	Detection of protruding lesions	COLON2	1	24	3640	860	Xception	Frame labeling of all datasets (normal vs. protruding lesions: polyps, epithelial tumors, subepithelial lesions)	Train–test split (80–20%)	Protruding lesions	91	93	97
	Ribeiro, 2022 [51]	Detection of ulcerative lesions	COLON2	2	124	37,319	3570	Xception	Frame labeling of all datasets (normal vs. ulcer or erosions)	train–validation (for hyperparameter tuning)–test split (70–20–10%)	Ulcers or erosions	97	100	100
	Majtner, 2021 [52]	Panenteric (small bowel and colon) detection of ulcerative lesions	CROHN	1	38	77,744	2748	ResNet	Frame labeling of all datasets (normal vs. ulcer or erosions)	Train–validation–test (70–20–10%) with patient split	Ulcers or erosions	96	100	NK
	Ferreira, 2022 [53]	Panenteric (small bowel and colon) detection of ulcerative lesions	CROHN	2	59	24,675	5300	Xception	Frame labeling of all datasets (normal vs. ulcer or erosions)	Train–test split (80–20%)	Ulcers or erosions	98	99	100
	Saraiva, 2021 [54]	Detection of blood	COLON2	1	24	5825	2975	Xception	Frame labeling of all datasets (normal vs. blood or hematic residues)	Train–test split (80–0%)	Blood or hematic residues	100	93	100
**Complex CNN for Enteric and Colonic Lesions**	Ding, 2019 [55]	Detection of abnormal findings in the small bowel without discrimination capacity	NaviCam	77	1970	158,235 + validation set	NK	ResNet	Frame labeling of training set (inflammation, ulcer, polyps, lymphangiectasia, bleeding, vascular disease, protruding lesion, lymphatic follicular hyperplasia, diverticulum, parasite, normal)	Testing with 5000 independent CE videos	Abnormal findings	100	100	NK
	Aoki, 2021 [56]	Detection of multiple types of lesions in the small bowel	SB3	3	NK	66,028 + validation set	44,684	Combined 3 SSD + 1 ResNet	Manual annotation of all mucosa breaks, angiectasias, protruding lesions and blood contents	CNN is trained on mixed data with positive and negative frames (44,684 lesions and 21,344 normal frames) Validation on 379 full videos	Mucosal brakes vs. other lesions	96	99	NK
											Angiectasias vs. other lesions	79	99	NK
											Protruding lesions vs. other lesions	100	95	NK
											Blood content vs. other lesions	100	100	NK
	Saraiva, 2021 [57]	Detection of multiple types of lesions in the small bowel + categorization of bleeding potential	SB3 OMON	2	5793	53,555	35,545	Xception	Frame labeling of all data (normal (N) vs. lymphangiectasias (P0L) vs. xanthomas (P0X) vs. erosions (P1E) vs. ulcers with uncertain/intermediate bleeding potential (P1U) vs. ulcers with high bleeding potential (P2U) vs. red spots (P1RS) vs. vascular lesions (angiectasias or varices) (P2V) vs. protruding lesions with uncertain/intermediate bleeding potential (P1P) vs. protruding lesions with high bleeding potential (P2P) vs. blood or hematic residues)	Train–test split (80–20%) with 11 × 11 confusion matrix	N vs. all	92	96	99
											P0L vs. all	88	99	99
											P0X vs. all	85	98	99
											P1E vs. all	73	99	97
											P1U vs. all	81	99	99
											P2U vs. all	94	98	100
											P1RS vs. all	80	99	98
											P2V vs. all	91	99	100
											P1P vs. all	93	99	99
											P2P vs. all	94	100	99
											Blood vs. all	99	100	100
	Saraiva, 2022 [58]	Detection of pleomorphic lesions or blood in the colon	COLON2	2	124	9005	pl5,930	Xception	Frame labeling of all datasets (normal (N) vs. blood or hematic residues (B) vs. mucosal lesions (ML), including ulcers, erosions, vascular lesions (red spots, angiectasia and varices) and protruding lesions (polyps, epithelial tumors, submucosal tumors and nodes))	Train–test split (80–20%) with 3 × 3 confusion matrix	N vs. all	97	96	100
											Blood vs. all	100	100	100
											ML vs. all	92	99	90
	Xie, 2022 [59]	Detection of multiple types of lesions in the small bowel + differentiation from normal mucosa	OMON	51	5825	757,770	NK	EfficientNet + Yolo	Frame labeling of all datasets Protruding lesions (venous structure, nodule, mass/tumor, polyp(s)), flat lesions (angiectasia, plaque (red), plaque (white), red spot, abnormal villi), mucosa (lymphangiectasia, erythematous, edematous), excavated lesion (erosion, ulcer, aphtha) and content (blood, parasite)	CNN is trained on mixed data with positive and negative frames Validation on 2898 full videos	Venous structure vs. all	98	100	NK
											Nodule vs. all	97	100	NK
											Mass or tumor vs. all	95	100	NK
											Polyp vs. all	95	100	NK
											Angiectasia vs. all	96	100	NK
											Plaque (red) vs. all	94	100	NK
											Plaque (white) vs. all	95	100	NK
											Red spot vs. all	96	100	NK
											Abnormal villi vs. all	95	100	NK
											Lymphangiectasia vs. all	98	100	NK
											Erythematous mucosa vs. all	95	100	NK
											Edematous mucosa vs. all	NK	NK	NK
											Erosion vs. all	NK	NK	NK
											Ulcer vs. all	NK	NK	NK
											Aphtha vs. all	NK	NK	NK
											Blood vs. all	NK	NK	NK
											Parasite vs. all	NK	NK	NK
**Complex CNN for Gastric Lesions**	Xia, 2021 [60]	Detection of multiple types of lesions + differentiation from normal mucosa	NaviCam MCE	1	797	1,023,955	NK	ResNet	Frame labeling of training set (erosions, polyps, ulcers, submucosal tumors, xanthomas, normal)	testing with 100 independent CE videos	Pleomorphic lesions	96	76	84
	Pan, 2022 [61]	Detect in real time of both gastric anatomic landmarks and different types of lesions	NaviCam MCE	1	906	34,062 + validation set	NK	ResNet	Frame labeling of all datasets (ulcerative (ulcer and erosions), protruding lesions (polyps and submucosal tumors), xanthomas, normal mucosa)	Prospective validation on 50 CE exams	Gastric lesions	99	NK	NK
											Anatomic landmarks	94	NK	NK
	Saraiva, 2023 [62]	Detection of pleomorphic gastric lesions	SB3 CROHN OMON	2	107	12,918	6074	Xception	Frame labeling of all datasets (normal vs. pleomorphic lesion (vascular, ulcerative or protruding lesion or blood/hematic residues))	Train–test split (80–20%) with patient split design and 3-fold cross-validation during training set	Pleomorphic lesions (mean of cross-validation)	88	92	96
											Pleomorphic lesions (test set)	97	96	100
